# 

**Published:** 1924-11

**Authors:** 


### HOSPITAL and HEALTH

REVIEW

*Established 1886* No. 1880. *Vol. LXXIII*

*New Series.* Vol. III. No. 38. *November,* 1924. Price Sixpence

CONTENTS

| | PAGE. |
|---|---|
| The Vindication of Voluntaryism | 327 |
| Preservatives and Food | 328 |
| Notes and Comments | 329 |
| Co-Education and St. Mary's—Improving Health Week—Spiritual Healing—The Best Investment—Healthy London—"Ignorant Aestheticism"—First Year's Work of Maudsley Hospital—Divided Councils—Hospitals and Blindness—Apt Adulteration's Artful Aid—Examining Policy Holders—Health and Penmanship—Congested Asylums—Artificial Light Treatment—Malaria in Corsica—An American View of Sanitary Work | |
| Hospital Men of Mark: Mr. Reginald B. Loder and Mr. Carrington S. Risbee | 333 |
| Liverpool Babies | 334 |
| Middlesex Hospital Prizegiving | 334 |
| Recovery of the Voluntary Hospitals | 335 |
| Wireless and Health | 336 |
| Lack of Food Standards. By Dr. Hutt, M.O.H. of Holborn | 337 |
| Nurses for Public Health Work | 338 |
| The Removal of Bethlem Hospital | 339 |
| A Sprightly Acrobat | 340 |
| North Ormesby Hospital Enlarged | 341 |
| Correspondence | 341 |
| The School in the Sun | 342 |
| Artificial Heat in Hospitals | 344 |
| Hospital Progress and Finance | 345 |
| Welfare Work in the Malay States | 346 |
| Alton Cottage Hospital Extension | 347 |
| Training of Nurses in Cottage Hospitals | 347 |
| Health Week at Middleton | 347 |
| Homœopathy at Bristol | 348 |
| Reviews of Books | 349 |
| Echoes of the Month | 353 |
| Points from Hospital Reports | 355 |
| Hospital and Health News | 357 |
| Recent Appointments | 358 |
| Answers to Correspondents | 358 |

